# Editorial: Mental health promotion and protection

**DOI:** 10.3389/fpsyt.2023.1161358

**Published:** 2023-02-27

**Authors:** Naseem Akhtar Qureshi, Samrat Singh Bhandari, Giorgio Di Lorenzo, Harshavardhan Sampath

**Affiliations:** ^1^Psychiatry Department, School of Medical Science and Research Center, Alfalah University, Faridabad, Haryana, India; ^2^Department of Psychiatry, Sikkim Manipal Institute of Medical Sciences, Sikkim Manipal University, Tadong, Sikkim, India; ^3^Department of Systems Medicine, University of Rome Tor Vergata, Rome, Italy

**Keywords:** promotion, protection, mental health, wellbeing, multifactorial

No man is an island, entire of itself;

Every man is a piece of the continent, a part of the main……!

—John Donne's (1572–1631) in Devotions upon Emergent Occasions.

Those lines penned by John in 1624 succinctly captured the challenges and opportunities facing not only mental health (MH) professionals but also other health experts worldwide. Like other horrifying pandemics of the past, the COVID-19 was a wake-up call that reminded all health professionals to seriously think of humanity as “a whole greater than the sum of its parts,” borrowing from the wisdom and research work of Gestalt psychologists who followed his steps. As clinicians who tirelessly work to ease the suffering of people with mental and physical disorders, we are often plagued by the nagging question at the back of our minds/psyche, “is this all I can do?” given the interconnected and multisectoral web of challenging predisposing, precipitating, and perpetuating factors in terms of biopsychosocial, epigenetic, and spiritual spanning the entire macrocosm often beyond our reach, as also evident in World Health Organization report (1). More specifically, a variety of factors that contribute toward good MH include civil liberties, politics, economics, social rights, and cultural diversity, and quality of life of the individual and the society as a whole. The promotion of mental health needs multidisciplinary approach as to cover all these important factors including education, work, justice, housing, human rights, and welfare and climate change (1, 2). In other words, the identification of barriers to mental health promotion and protection also is of paramount importance which could be addressed at various levels including family, community, society and general population.

However, we are reminded by the lines in the movie Matrix (Reloaded), “Hope. It is the quintessential human delusion, simultaneously the source of (y) our greatest strength, and greatest weakness.” Born of this delusional—like hope, this Research Topic on “*Mental health promotion and protection*” was launched by the Frontiers Psychiatry in July 2021. The topic of research was selected by Dr. Qureshi NA and Frontiers' Chief Executive Officer. We are overwhelmed by the positive response to our initiative-positive mental, physical, and social health, and its protection and promotion associated with wellbeing and resilience of people globally-evidenced by high quality, ingenuity and creativity, and breath of research manuscripts submitted. This Research Topic is highlighted by research on divergently connected areas in the vast terrain-biopsychosociocultural-of MH prevention, promotion and protection.

The research by Tian et al., on the effort-reward imbalance in ER physicians, the review on MH interventions at the workplace by Pandya et al. and the impact of welfare schemes and programs offering fringe benefits to urban and rural employees by Luo et al. span the multiple areas of occupational MH.

The MH of students and young adults is captured by Hu J et al., in their study on MH literacy, sleep and depressive symptoms. A worldwide online survey of 65 countries on stigma of medical students toward psychiatric patients by Babicki et al., shows that more needs and efforts to be done and address this pernicious attitude.

The MH of female domestic workers and their relation to social networks by Tang et al., and the MH of Trans women and its association with sexual myths by Uyar et al., explore issues in marginalized and minority populations.

Technology-driven MH promotion is presented with positive results by Wu J et al., in their study of mindfulness exercise guided by a Smartphone application. Gatto et al., have presented positive results concerning an online brief emotional training program for emerging adults, and Kim et al., have developed an online-coaching blended couple-oriented intervention for adults. At the same time, the negative effects of technology on MH have been explored by Zhang et al., on smartphone addiction, sleep, academic stress, and depression.

Promoting the adherence to treatment in chronic and severe mental disorders, Sun et al., investigated the factors influencing the attitudes of patients living in the community toward long-acting depot antipsychotics, while the risk factors of violent behavior in schizophrenic psychosis is presented by Huang et al., in a 2-year follow-up study. Interestingly, wisdom as a protective factor for psychotic-like experiences in the general population are reported by Wu Z et al. Sexuality in people with ultra-high risk such as late adolescents and early adulthood, genetic vulnerability and attenuated psychotic symptoms and first-episode psychosis is systematically reviewed by Ciocca et al..

In the area of liaison psychiatry, depression, type 2 diabetes mellitus, and coronary atherosclerotic heart disease by Li W et al.; chronic low back pain and depression by Hu Y et al.; body image concerns in hemodialysis by Nia et al.; social support and quality of life in female systemic lupus erythromatosis sufferers by Gao et al.; the impact of maternal depression on neonatal outcomes by Li H et al.; psychological factors affecting treatment adherence in patients with tuberculosis by Pradhan et al.; and finally a review of the impact of MH on outcome in tuberculosis by Agbeko et al., are presented in this issue.

Novel studies including the research by Chen et al., on the restorative effect of rooftop gardens in urban settings needs special mention. Similarly, Zhou et al., have demonstrated that childhood neighborhood cohesion has a significant impact on neurocognitive functions in later life. Also a protocol for assessing the feasibility of psychological or MH first aid training of front-line healthcare workers in the provision of MH care during emergencies is presented by Wang L et al..

The role of community services on positive MH is highlighted by Yang et al., in their study on the impact of volunteering services in older adults and Wang J et al., in their research paper related to social participation in community activities in middle age and elderly residents.

Finally, the impact of the COVID-19 pandemic on the MH of healthcare workers in Italy during the reopening phase after lockdown by Moro et al., and the review of telemental health use during COVID-19 pandemic by Abraham et al., find place in this Research Topic.

To summarize, we are sure this issue does justice to MH Promotion and Protection, as we coordinate our resources, influence policy makers, engage with the community, and strive to make this World a better place for our future generations. We tentatively suggest the next topic of greatest interest: “Traumatic events in infants and adolescents and its psychiatric trajectories in future life.”

## Author contributions

All authors listed have made a substantial, direct, and intellectual contribution to the work and approved it for publication.
